# Image-based modelling of nutrient movement in and around the rhizosphere

**DOI:** 10.1093/jxb/erv544

**Published:** 2016-01-05

**Authors:** Keith R. Daly, Samuel D. Keyes, Shakil Masum, Tiina Roose

**Affiliations:** ^1^Bioengineering Sciences Research Group, Faculty of Engineering and Environment, University of Southampton, University Road, SO17 1BJ Southampton, UK; ^2^School of Energy, Environment and Agrifood, Cranfield University, Cranfield, MK43 0AL, UK

**Keywords:** Phosphate, plant–soil interaction, rhizosphere, structural imaging, X-ray CT.

## Abstract

In this study, we developed a spatially explicit model for nutrient uptake by root hairs based on X-ray computed tomography images of the rhizosphere soil structure. This work extends our previous work to larger domains and hence is valid for longer times. Unlike the model used previously, which considered only a small region of soil about the root, we considered an effectively infinite volume of bulk soil about the rhizosphere. We asked the question: At what distance away from root surfaces do the specific structural features of root-hair and soil aggregate morphology not matter because average properties start dominating the nutrient transport? The resulting model was used to capture bulk and rhizosphere soil properties by considering representative volumes of soil far from the root and adjacent to the root, respectively. By increasing the size of the volumes that we considered, the diffusive impedance of the bulk soil and root uptake were seen to converge. We did this for two different values of water content. We found that the size of region for which the nutrient uptake properties converged to a fixed value was dependent on the water saturation. In the fully saturated case, the region of soil we needed to consider was only of radius 1.1mm for poorly soil-mobile species such as phosphate. However, in the case of a partially saturated medium (relative saturation 0.3), we found that a radius of 1.4mm was necessary. This suggests that, in addition to the geometrical properties of the rhizosphere, there is an additional effect of soil moisture properties, which extends further from the root and may relate to other chemical changes in the rhizosphere. The latter were not explicitly included in our model.

## Highlight

Rigorous mathematical techniques and image-based modelling were used to quantify the effect of root hairs on nutrient uptake and to investigate how uptake is influenced by growing root hairs.

## Introduction

The uptake of phosphate and other low-mobility nutrients is essential for plant growth and hence global food security (Nye and Tinker, 1977; Barber, 1984; Tinker and Nye, 2000). However, the overuse of inorganic fertilizers has caused an accumulation of phosphate in European soils, which potentially causes eutrophication (Gahoonia and Nielsen, 2004). Hence, there is a need to optimize the efficiency of phosphate uptake not only to increase crop yields but also to minimize the detrimental effects of excess phosphate on the environment.

Plants are known to uptake nutrients through both their roots and root hairs (Datta *et al.*, 2011). Root hairs are thought to play a significant role in the uptake of poorly soil-mobile nutrients such as phosphate, and in water uptake, plant stability, and microbial interactions (Gahoonia and Nielsen, 2004; Datta *et al.*, 2011). In order to quantify the role of root hairs in the uptake of poorly soil-mobile nutrients, such as phosphate, a detailed understanding of the root-hair morphology and the region of soil around the root, known as the rhizosphere (Hiltner, 1904), is required. This is because the properties of soil in the rhizosphere are thought to be significantly different due to soil–microbial interactions and compaction of soil by the root (Dexter, 1987; Whalley *et al.*, 2005; Aravena *et al.*, 2010, 2014; Daly *et al.*, 2015).

Early models for nutrient movement in soil treated the rhizosphere as a volume-averaged continuum (Nye and Tinker, 1977; Barber, 1984; Ma *et al.*, 2001). More recently, models for nutrient movement have been derived and parameterized for dual porosity soil (Zygalakis *et al.*, 2011) and for soil adjacent to cluster roots (Zygalakis and Roose, 2012). These models were based around the technique of homogenization (Hornung, 1997; Pavliotis and Stuart, 2008), a multi-scale technique that enables field-scale equations to be derived and parameterized based on underlying continuum models.

To obtain a better understanding of processes occurring in the rhizosphere, in comparison with the surrounding bulk soil, non-invasive measurements of the plant root and soil structure are essential (Hallett *et al.*, 2013; George *et al.*, 2014). Three-dimensional imaging of plant roots *in situ* using X-ray computed tomography (CT) is a rapidly growing field (Tracy *et al.*, 2010; Aravena *et al.*, 2010, 2014; Hallett *et al.*, 2013; Keyes *et al.*, 2013). Using these techniques, submicron resolutions can be achieved enabling the root-hair morphology to be visualized (Keyes *et al.*, 2013).

In addition to direct *in situ* visualization of plant–soil interaction, the use of X-ray CT also provides the means to apply numerical models describing the diffusion of nutrients directly to the imaged geometries (Aravena *et al.*, 2010, 2014; Keyes *et al.*, 2013; Daly *et al.*, 2015; Tracy *et al.*, 2015). This approach has been used to quantify the effectiveness of root hairs in saturated soil conditions (Keyes *et al.*, 2013) using a diffusion model originally developed for mycorrhizal fungi (Schnepf *et al.*, 2011). However, the study of Keyes *et al.* (2013) considered a small volume of soil adjacent to the root. This volume extended approximately 300 μm from the root surface and had a zero flux boundary condition on the outer surface. Hence, once the phosphate immediately adjacent to the root was depleted, the uptake of nutrients into the root stopped. This, while accurate for the experimental situation considered, limited the time frame for which the model was applicable to around 3h.

In this study, we extended the work of Keyes *et al.* (2013) to include both bulk and rhizosphere soil. The key aims were to develop an image-based modelling approach that was applicable to root hairs surrounded by a large/infinite bulk of soil, to understand how root hairs contribute to nutrient uptake at different soil water contents and to estimate the effects of root-hair growth on nutrient uptake. We chose to model a single root in a large/infinite volume of soil, rather than multiple roots competing for nutrients for a number of reasons. First, we wanted to compare our model with established models for nutrient uptake that are applied in this geometry (Roose *et al.*, 2001). Secondly, while some models consider root–root competition (Barber, 1984), these models were applied in a cylindrical geometry. Hence, any competition was provided by a ring of roots at a distance from the root under consideration. In order to accurately capture roots at a given root density, additional assumptions and approximations would be required. Finally, our aim was to study the role of root hairs in the uptake of a single root. Hence, we chose to study a geometry that captures and isolates this effect. The modelling strategy developed in this study will provide a framework for future modelling studies to consider how different root-hair morphologies influence uptake. In addition, it will provide a new level of understanding of the role of root hairs in nutrient uptake as the root system begins to develop.

The bulk soil properties used in this study were derived using multi-scale homogenization combined with image-based modelling in a similar manner to Daly *et al.* (2015). The resulting equations were solved analytically and were patched to an explicit image-based geometry of the rhizosphere using a time-dependent boundary condition. Reducing the bulk soil to a single boundary condition allowed us to capture the full geometry and topology of the plant–root–soil system without the need for excessive computational resources and has particular relevance to poorly soil-mobile species such as phosphate, potassium, and zinc. The model describes phosphate uptake by roots and root hairs in an effectively infinite volume of bulk soil while capturing at high precision any changes in the soil adjacent to the plant roots. In this case, an effectively infinite volume of soil corresponds to any volume of soil that is sufficiently large that the phosphate depletion region about the root does not reach the edge of the domain considered. Using this model, we were able to parameterize upscaled models for nutrient motion in soil (Zygalakis *et al.*, 2011).

This report is arranged as follows: we first describe the plant growth, imaging, and modelling approaches, and then discuss the results of numerical simulation and show how these models can be used to perform an image-based study of root-hair growth; finally, we discuss our results and show how these models might be further developed. The technical description of the mathematical models used in this paper is provided in Supplementary data A and B (available at *JXB* online).

## Methods

### Plant growth

We studied the rice genotype *Oryza sativa* cv. Varyla provided by the Japan International Research Centre for Agricultural Sciences. The seeds were heat treated at 50 °C for 48h to break dormancy and standardize germination. Germination was carried out at 23±1 °C for 2 d between moistened sheets of Millipore filter paper. The growth medium was a sand-textured Eutric Cambisol soil collected from a surface plot at Abergwyngregyn, North Wales, UK (53°14′N, 4°01′W), and the soil organic matter was 7% (full details are given by Lucas and Jones, 2006, soil B). The soil was sieved to <5mm, autoclaved, and air dried at 23±1 °C for 2 d. The dried soil was sieved to between bounds of 1680 and 1000 μm, producing a well-aggregated, textured growth medium.

Growth took place in a controlled growth environment (Fitotron SGR; Weiss-Gallenkamp, Loughborough, UK) for a period of 14 d. Growth conditions were 23±1 °C and 60% humidity for 16h (day) and 18±1 °C and 55% humidity for 8h (night) with both ramped over 30min.

The seminal roots were guided by a specially designed growth environment fabricated in ABS plastic using an UP! 3D printer (PP3DP, China). The resulting assay is shown in Supplementary Fig. S1 (available at *JXB* online). The morphology of the growth environment was used to guide the roots into seven syringe barrels (root chambers), which could then be detached for imaging once the growth stage was complete. The lower portion including the root chambers was housed in a foil-wrapped 50ml centrifuge tube, which occluded light during the growth period.

### Imaging

Imaging of root chambers was conducted at the TOMCAT beamline on the X02DA port of the Swiss Light Source, a third-generation synchrotron at the Paul Scherrer Institute, Villigen, Switzerland. The imaging resolution was 1.2 μm, and a monochromatic beam was employed with an energy of 19kV. A 90ms exposure time was employed to collect 1601 projections over 180° with a total scan time of 2.4min. The data were reconstructed to 16-bit volumes using a custom back-projection algorithm implementing a Parzen filter. The resulting volume size was 2560×2560×2180 voxels.

The reconstructed volumes were analysed using a multi-pass approach (S.D. Keyes, K. Zygalakis, T. Roose, unpublished data). While root hairs can be visualized *in situ* for dry soils, they are challenging to distinguish from some background phases, even at high resolution. Specifically, the portions of root hairs that traverse water-filled regions of the pore space are indistinguishable from the water. The visible sections of the root hairs were extracted manually using a graphical input tablet (Cintiq 24; WACOM) and a software package that allowed interpolation between lofted cross-sections (Avizo FIRE 8; FEI Company, OR, USA). A number of these sections represent an incomplete root-hair segmentation since proportions of the hair paths can be occluded by fluid-rich phases and pore water. Full root-hair paths were extrapolated from the segmented sections of partially visible hairs using an algorithm that simulates root-hair growth (S.D. Keyes, K. Zygalakis, T. Roose, unpublished data). The algorithm was parameterized from the root hairs of plants (*O. sativa* cv. Varyla), which were grown in rhizo-boxes with dimensions 30×30×2cm, constructed of transparent polycarbonate with a removable front plate, and wrapped in foil to occlude light. Hydration during the growth period was maintained via capillary rise from a tray of water, with occasional top watering. The plants were grown in a glasshouse, with temperatures ranging from 20–32 °C over the growth period. The root systems of 1-month-old specimens were gently freed from soil using running water. One fine primary and one coarse primary were randomly sampled before manual removal of the remaining soil from the roots. Each was cut into a number of subsections of 2cm length for analysis, and each cut was imaged using an Olympus BX50 optical microscope. After stitching all images together, five subregions were randomly defined for each section. Each region was chosen to represent a distance of 1mm on the root surface. All hairs in subregions that were in sharp focus were measured in FIJI using a poly-line tool to generate a set of 220 hair lengths. A normal distribution was fitted to these data and used to parameterize the hair-growth algorithm. The fitted and sampled distributions are shown in Supplementary Fig. S1. The mean of experimental hair length was 171.56 µm, compared with the mean fitted lengths of 173.67 µm. The standard deviation of the experimental hair length was 72.88 µm compared with the standard deviation of the fitted length of 73.44 µm.

We observe that root-hair density measured via synchrotron radiation X-ray CT data was generally slightly higher than for microscopy measurements, probably due to the lack of damage artefacts. For the data in this study, the density of hairs was approximately 77 hairs mm^–1^ compared with 64 hairs mm^–1^ measured via bright-field microscopy in a study of similar rice varieties grown under similar conditions (Wissuwa and Ae, 2001). This discrepancy was, in part, because the *in situ* hairs were often part-occluded by fluid and colloidal phases, and it is usually only possible to obtain partial measurements in the synchrotron CT data. This was offset to some degree by the lack of damage artefacts when compared with root washing and microscopy, but the length values were low compared with most literature sources. However, in this study, we explored the implications of soil domain size on nutrient uptake and used hair data attained via quantification of soil-grown roots using bright-field microscopy.

The root geometry was extracted in the same manner, manually defining the root cross-section from slices sampled (at a spacing of 120 µm) along the root growth axis. The soil minerals, pore gas, and pore fluid were extracted using a trainable segmentation plug-in in FIJI that implements the WEKA classifiers for feature detection using a range of local statistics as training parameters (Hall *et al.*, 2009).

The segmented geometry was used to produce three-dimensional surface meshes, using the ScanIP software package (Simpleware Ltd, Exeter, UK). The root, hairs, gas phase, fluid phase, and soil minerals were exported as separate STL files, which provided the geometries on which the numerical models could be tested.

### Modelling

**Fig. 1.**
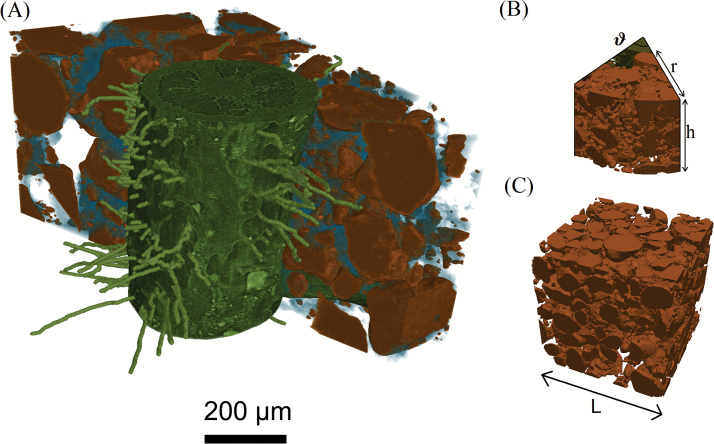
Image-based geometry: (A) X-ray CT image of the roots, root hairs, soil, and pore water. (B) One of the rhizosphere segments considered with the size described by the angle θ, height *h*, and radius *r*. (C) A cube of bulk soil of side length *L*. (This figure is available in colour at *JXB* online.)

We modelled the uptake of phosphate by a single root and root hairs in an unbounded partially saturated geometry (Fig. 1). In order to do this, we extended the model described by Keyes *et al.* (2013) in which the authors considered a segment of root and surrounding soil. On the outer boundary of the segment, a zero flux condition was applied. By definition, this condition prevents any influx of nutrient from outside the domain considered. Hence, the total possible uptake is limited to the nutrient available from within the initial segment. This was adequate for the experimental situation considered, i.e. a pot of radius ~300 μm, but is clearly not applicable for isolated roots in soil columns of radius larger than 300 μm. We noted that this is a slightly different scenario from the one studied by Barber (1984) in which the domain around the root was considered to be half the size of the inter-root spacing. In other words, Barber’s model considered multiple roots, thereby effectively introducing root competition. In this study, we consider a single isolated root and how this root was affected by the soil structural properties.

In order to overcome this limitation, we considered a single root in a large/infinite domain of soil. However, as considering an infinite domain explicitly is computationally unfeasible, we broke the domain into two regions: the rhizosphere and the bulk soil. We assumed that the bulk soil, which was far from the root, might be considered as a homogeneous medium with an effective phosphate diffusion constant. The effective diffusion properties of phosphate in the bulk soil were derived from the segmented CT data using the method of homogenization (Pavliotis and Stuart, 2008; Zygalakis *et al.*, 2011; Zygalakis and Roose, 2012). The nutrient flux in the bulk soil was then patched to the rhizosphere domain using a boundary condition that simulated an effective infinite region of bulk soil beyond the rhizosphere. In this section, we introduce the equations and summarize the model that resulted from these assumptions. Full details of the derivation have been included in Supplementary data A and B.

**Fig. 2.**
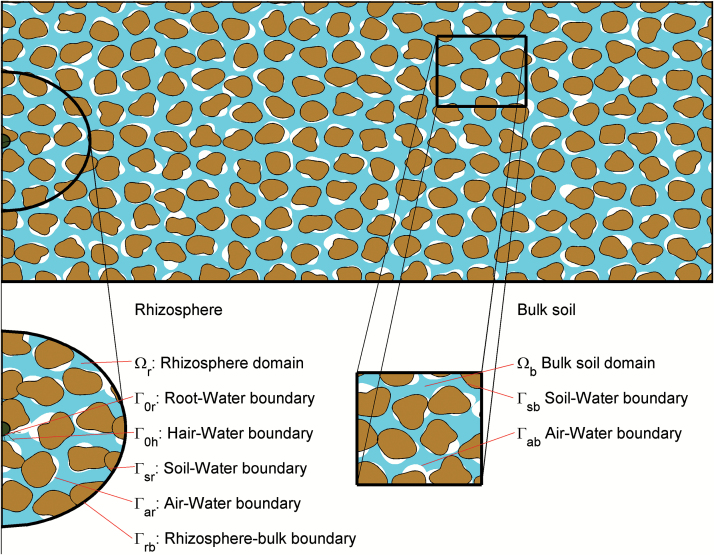
Schematic of the different regions used in the mathematical formulation of the problem. The bulk and rhizosphere regions are highlighted with the modelling domains $\Omega_{r},$ $\Gamma_{0r},$ $\Gamma_{0h},$ $\Gamma_{sr},$ $\Gamma_{ar},$ and $\Gamma_{rb}$labelled for the rhizosphere and $\Omega_{b},$ $\Gamma_{sb}$and $\Gamma_{ab}$labelled for the bulk soil. (This figure is available in colour at *JXB* online.)

We assumed that phosphate could only diffuse in the fluid domain, i.e. no diffusion occurred in the air-filled portion of the pore space. Therefore, we only present equations in the fluid region and its associated boundaries. However, we made no assumptions about the geometrical details regarding the air-filled portion of pore space. If, from the CT data, there was air in contact with soil or root material, this simply acted to reduce the surface area over which phosphate could be exchanged. We defined two regions of fluid-filled pore space. We denoted the rhizosphere by $\Omega_{r}$ as the region explicitly considered near to the root. Physically, we may think of this as an approximation to the rhizosphere. The second region was $\Omega_{b}$, the region of bulk soil outside of the rhizosphere (Fig. 2). As different boundary conditions need to be applied on each of the pore–water interfaces, we adopted the following naming convention. We took the interface between the two regions, located at a distance ${\tilde{r}}_{b}$ from the centre of the root, to be $\Gamma_{rb}.$The root and root-hair surfaces were defined as $\Gamma_{0r},$and $\Gamma_{0h},$respectively, and the soil particle surface to be $\Gamma_{sr}$and $\Gamma_{sb}$in regions $\Omega_{r}$and $\Omega_{b},$respectively. Finally, we defined the air–water interfaces as $\Gamma_{ar}$ and $\Gamma_{ab}$ in regions $\Omega_{r}$ and $\Omega_{b}$, respectively. The method we used to determine phosphate movement was different in each region. Phosphate movement in the rhizosphere was calculated based on a spatially explicit model obtained from the CT data. Phosphate movement in the bulk soil was calculated analytically based on the solution to a spatially explicit numerical model in a representative volume of bulk soil. We present each of these models separately below.

#### Rhizosphere

In the rhizosphere, we assumed that phosphate moves by diffusion only:

$$\frac{\partial {\tilde{C}}_{r}}{\partial \tilde{t}}=\tilde{D}{\tilde{\nabla }}^{2}{\tilde{C}}_{r},\;x\in \Omega_{r},$$ (1)

where ${\tilde{C}}_{r}$ is the phosphate concentration in the soil solution and $\tilde{D}$ is the diffusion constant of phosphate in the soil solution. The phosphate was assumed to bind to the soil particles based on linear first-order kinetics:

$$\tilde{D}\hat{n}\cdot \tilde{\nabla }{\tilde{C}}_{r}=-{\tilde{k}}_{a}{\tilde{C}}_{r}+{\tilde{k}}_{d}{\tilde{C}}_{a},\;x\in \Gamma_{sr},$$ (2)

$$\frac{\partial {\tilde{C}}_{a}}{\partial \tilde{t}}={\tilde{k}}_{a}{\tilde{C}}_{r}-{\tilde{k}}_{d}{\tilde{C}}_{a},\;x\in \Gamma_{sr},$$ (3)

where ${\tilde{k}}_{a}$ and ${\tilde{k}}_{d}$ are the adsorption and desorption rates respectively, ${\tilde{C}}_{a}$ is the nutrient concentration on the soil surface, and$\hat{n}$is the outward pointing surface normal vector. Here, we have assumed that the nutrient concentration of the soil can be represented as a surface concentration and that any replenishment from within the soil aggregate is either so fast that it is captured by these equations or so slow that we can neglect it. We assumed that there was no nutrient diffusion across the air–water boundary:

$$\tilde{D}\hat{n}\cdot \tilde{\nabla }{\tilde{C}}_{r}=0,\;x\in \Gamma_{ar}.$$ (4)

We considered uptake of phosphate from the fluid only. On the root surface, this followed a linear uptake condition:

$$\tilde{D}\hat{n}\cdot \tilde{\nabla }{\tilde{C}}_{r}=\tilde{\lambda }{\tilde{C}}_{r},\;x\in \Gamma_{0r},$$ (5)

where $\tilde{\lambda }$ is the nutrient uptake rate on the root and root-hair surfaces. For comparison with the paper by Keyes *et al.* (2013), we chose the absorbing power as $\tilde{\lambda }=\frac{{\tilde{F}}_{m}}{{\tilde{K}}_{m}}$, consistent with Nye and Tinker. Equation (5) is the small concentration equivalent of the Michaelis–Menten condition, in which ${\tilde{F}}_{m}$ is the maximum rate of uptake and ${\tilde{K}}_{m}$ is the concentration when the uptake is half the maximum.

The flux on the root-hair surface was given by one of two different scenarios. The first was linear uptake:

$$\tilde{D}\hat{n}\cdot \tilde{\nabla }{\tilde{C}}_{r}=\tilde{\lambda }{\tilde{C}}_{r},\;x\in \Gamma_{0h}.$$ (6)

**Fig. 3.**
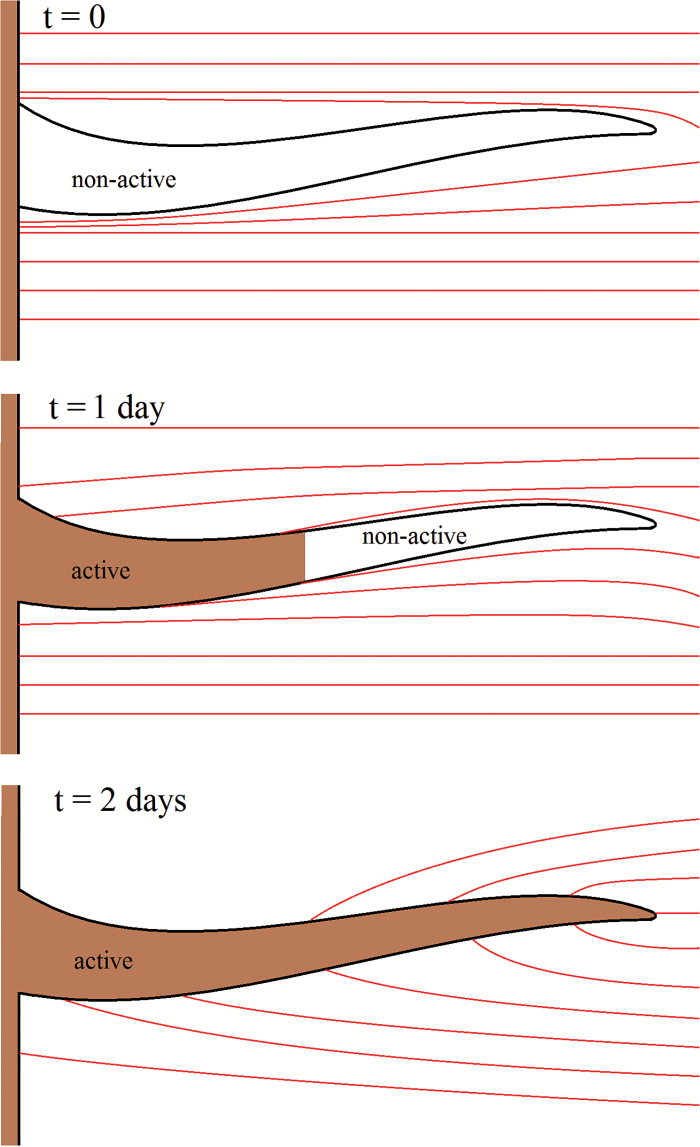
Schematic for root-hair growth. The root-hair geometry remains unchanged throughout the simulation. As the simulation progresses, the active portion of the root hair grows. The streamlines show an estimation of the nutrient transport as affected by the presence of the root hair. At *t*=0, the root hair is non-active and acts only to geometrically impede the transport of nutrient. At *t*=1 d, the root hair has grown to half its final length and acts partly to take up nutrients and partly as a geometrical impedance. Finally, at *t*=2 d, the root hair is fully grown. (This figure is available in colour at *JXB* online.)

The second case we considered was a pseudo time-dependent uptake, which simulates linear uptake for a growing root-hair. We termed this pseudo root-hair growth, as we did not consider the geometrical and mechanical effects of root-hair growth explicitly. Rather, we assumed that at $\tilde{t}=0$ the root hairs are present but do not contribute to uptake. As the simulation progresses, the root hairs are assumed to grow at a certain rate. We modelled this by assuming an active region of root hair, which grows outward from the root; this is illustrated in Fig. 3. We write the root-hair uptake condition as:

$$\tilde{D}\hat{n}\cdot \tilde{\nabla }{\tilde{C}}_{r}=\frac{\tilde{\lambda }}{2}{\tilde{C}}_{r}\left\{1+\tanh\left[-\tilde{\alpha }\left(\tilde{r}-{\tilde{g}}_{r}\tilde{t}\right)\right]\right\},\;x\in \Gamma_{0h},$$ (7)

where $\tilde{r}$ is the axial distance along the root hair, ${\tilde{g}}_{r}$ is the root-hair growth rate, and $\tilde{\alpha }$ is an empirical parameter that controls the sharpness of the transition between the root hairs being ‘on’, i.e. actively taking up nutrient and ‘off’, i.e. not actively taking up nutrient.

The adsorption and desorption constants (${\tilde{k}}_{a}\;{\text{and }\tilde{k}}_{d}$) were taken from Keyes *et al.* (2013) where they were derived using standard soil tests (Murphy and Riley, 1962; Giesler and Lundström, 1993). The remaining parameters used in Eqns (1)–(5) were obtained from the literature (Barber, 1984; Tinker and Nye, 2000) and are summarized in Table 1.

**Table 1.** Physical parameters used in the nutrient model

Constant	Value	Units	Description
$\tilde{D}$	$10^{-9}$	${\text{m}}^{2}\;{\text{s}}^{-1}$	Diffusion constant
${\tilde{K}}_{m}$	$6\times 10^{-3}$	$\text{mol }{\text{m}}^{-3}$	Uptake constant
${\tilde{F}}_{m}$	$5\times 10^{-9}$	$\text{mol }{\text{m}}^{-2}s^{-1}$	Uptake rate
${\tilde{k}}_{d}$	$2.569\times 10^{-4}$	${\text{s}}^{-1}$	Desorption rate
${\tilde{k}}_{a}$	$3.73\times 10^{-9}$	$\text{m}\;s^{-1}$	Adsorption rate
${\tilde{g}}_{r}$	$5\times 10^{-9}$	$\text{m}\;s^{-1}$	Root-hair growth rate
$\tilde{\alpha }$	$0.2\times 10^{6}$	${\text{m}}^{-1}$	Root-hair cut off rate

#### Rhizosphere bulk interface

Due to the success of averaged equations in bulk soil, for example Darcy’s law and Richards’ equation (Van Genuchten, 1980), the precise details of the geometry are less important in determining the total phosphate movement and average properties are sufficient. Therefore, rather than consider the explicit details of the geometry, we derived an effective diffusion constant based on a representative soil volume in the absence of roots. This was achieved using the method of homogenization (Pavliotis and Stuart, 2008), which was implemented as follows. First, it was shown that, on the length scale of interest, the nutrient concentration was only weakly dependent on the precise structure of the soil. Secondly, a set of equations, often called the cell problem, were derived that determined the local variation in concentration due to the representative soil volume. Finally, an averaged equation was derived that described the effective rate of diffusion in terms of an effective diffusion constant ${\tilde{D}}_{eff}=\tilde{D}D_{eff}$, where $D_{eff}$ is calculated from the bulk soil geometry (Eqns A31–A34 and A58 in Supplementary data) and describes the impedance to diffusion offered by the soil. Full details of how $D_{eff}$ was derived are provided in Supplementary data A. Hence, in the bulk the diffusion of phosphate was described by:

$$\frac{\partial {\tilde{C}}_{b}}{\partial \tilde{t}}={\tilde{D}}_{eff}{\tilde{\nabla }}^{2}{\tilde{C}}_{b},$$ (8)

where ${\tilde{C}}_{b}$ is the nutrient concentration in the bulk soil. The advantage of Eqn (8) is that we do not need to explicitly consider the soil geometry in the bulk soil domain. In itself, this significantly reduces the computational cost for finite, but large, domains. However, rather than solving Eqn (8) numerically, we found an approximate analytic solution for an infinite bulk soil domain subject to the conditions ${\tilde{C}}_{b}={\tilde{C}}_{r}\left(t\right)$ for $x\in \Gamma_{rb}$ and ${\tilde{C}}_{b}\to {\tilde{C}}_{\infty }$ as $\tilde{r}\to \infty$. Using this solution, we were able to define a relationship between concentration and flux at the edge of the rhizosphere. The result was a condition that simulates the presence of an infinite region of bulk soil at the rhizosphere–soil domain boundary:

$$\tilde{D}\hat{n}\cdot \tilde{\nabla }{\tilde{C}}_{r}=-\frac{2{\tilde{D}}_{eff}({\tilde{C}}_{\infty }-{\tilde{C}}_{r})}{{\tilde{r}}_{b}\ln\left(\frac{4{\tilde{D}}_{eff}\tilde{t}}{{\tilde{r}}_{b}^{2}e^{-\gamma }}\right)\;},\;x\in \Gamma_{rb},$$ (9)

where γ=0.57721 is the Euler–Mascheroni constant. Hence, the bulk soil was dealt with entirely by the conditions in Eqn (9). This significantly reduced the computational cost while allowing us to include the averaged geometric details of the bulk soil through the parameter ${\tilde{D}}_{eff}.$We noted that the boundary condition in Eqn (9) was singular at $\tilde{t}=0.$This was regularized by the fact that when $\tilde{t}=0,$ we have ${\tilde{C}}_{\infty }={\tilde{C}}_{r}.$ To overcome the difficulties of implementing this, we followed the suggestion of Roose *et al.* (2001) and modified Eqn (9) such that for small $\tilde{t}$the equation was non-singular (Supplementary data B). To summarize, the final set of equations we solved in the rhizosphere were:

$$\frac{\partial {\tilde{C}}_{r}}{\partial \tilde{t}}=\tilde{D}{\tilde{\nabla }}^{2}{\tilde{C}}_{r},\;x\in \Omega_{r},$$ (10)

$$\tilde{D}\hat{n}\cdot \tilde{\nabla }{\tilde{C}}_{r}=-{\tilde{k}}_{a}{\tilde{C}}_{r}+{\tilde{k}}_{d}{\tilde{C}}_{a},\;x\in \Gamma_{sr},$$ (11)

$$\frac{\partial {\tilde{C}}_{a}}{\partial \tilde{t}}={\tilde{k}}_{a}{\tilde{C}}_{r}-{\tilde{k}}_{d}{\tilde{C}}_{a},\;x\in \Gamma_{sr},$$ (12)

$$\tilde{D}\hat{n}\cdot \tilde{\nabla }{\tilde{C}}_{r}=0,\;x\in \Gamma_{ar},$$ (13)

$$\tilde{D}\hat{n}\cdot \tilde{\nabla }{\tilde{C}}_{r}=\tilde{\lambda }{\tilde{C}}_{r},\;x\in \Gamma_{0r},$$ (14)

$$\tilde{D}\hat{n}\cdot \tilde{\nabla }{\tilde{C}}_{r}=\frac{-2{\tilde{D}}_{eff}({\tilde{C}}_{\infty }-{\tilde{C}}_{r})}{2{\tilde{r}}_{b}+{\tilde{r}}_{b}\left[\gamma -\ln\left(4{\tilde{D}}_{eff}\tilde{t}/{\tilde{r}}_{b}^{2}+e^{\gamma }\right)\right]\;}\;x\in \Gamma_{rb},$$ (15)

where, in the linear uptake case, the root-hair boundary condition was given by:

$$\tilde{D}\hat{n}\cdot \tilde{\nabla }{\tilde{C}}_{r}=\tilde{\lambda }{\tilde{C}}_{r},\;x\in \Gamma_{0h}.$$ (16)

In the root-hair growth scenario, the root-hair boundary condition was given by:

$$\tilde{D}\hat{n}\cdot \tilde{\nabla }{\tilde{C}}_{r}=\frac{\tilde{\lambda }}{2}{\tilde{C}}_{r}\left\{1+\tanh\left[-\tilde{\alpha }\left(\tilde{r}-{\tilde{g}}_{r}\tilde{t}\right)\right]\right\},\;x\in \Gamma_{0h},$$ (17)

and $\tilde{D}\hat{n}\cdot \tilde{\nabla }{\tilde{C}}_{r}=0$ was applied on the remaining external boundaries (Fig. 2.) Equations (10)–(17) describe the uptake of phosphate by roots and root hairs from an infinite bulk of soil.

Using the STL files generated from the CT data, a computational mesh was constructed using the snappyHexMesh package. The mesh was generated from a coarse hexahedral mesh and the STL surface meshes from the imaged geometry. The hexahedral mesh was shaped like a segment of initial angle $\tilde{\theta },$ height $\tilde{h},$ and radius ${\tilde{r}}_{b}$ corresponding to the size of the domain to be modelled. Each of these parameters was increased until the root uptake properties converged. Successive mesh refinements were made where the mesh intersected any geometrical feature described by the STL files, i.e. the mesh was refined about the surfaces of interest. Once the mesh had been refined to hexahedra of side length <1 μm about the STL surfaces, the regions outside of the water domain were removed. The remaining mesh was then deformed to match to the STL surfaces. Finally, the mesh was smoothed to produce a high-quality mesh on which the numerical models could be run. The equations are discretized on the mesh and solved in OpenFOAM, an open source finite volume code (Jasak *et al.*, 2013), using a modified version of the inbuilt LaplacianFOAM solver to couple the bulk concentration to the sorbed concentration at the soil boundaries. Time stepping was achieved using an implicit Euler method with a variable time-stepping algorithm for speed and accuracy. All numerical solutions were obtained using the Iridis 4 supercomputing cluster at the University of Southampton, UK.

## Results and discussion

Using the theory developed above, we calculated the bulk soil effective diffusion constant and nutrient uptake properties of a single root with hairs. We considered two different water contents. The first was the case in which all the pore space was full of water, i.e. *S*=1, where *S* is the volumetric water content defined as the volume of water divided by the volume of pore space. Secondly, we used the segmented CT image to obtain the water content and air–water interface from the scanned soil. In this case, the volumetric water content was *S*=0.33. In addition to being able to parameterize existing models, we also calculated the size of the region that needs to be considered for the uptake predicted by our simulations to converge. Finally, we considered how a growing root-hair system affects the overall uptake properties of the root and root hairs.

### Bulk soil properties

**Fig. 4.**
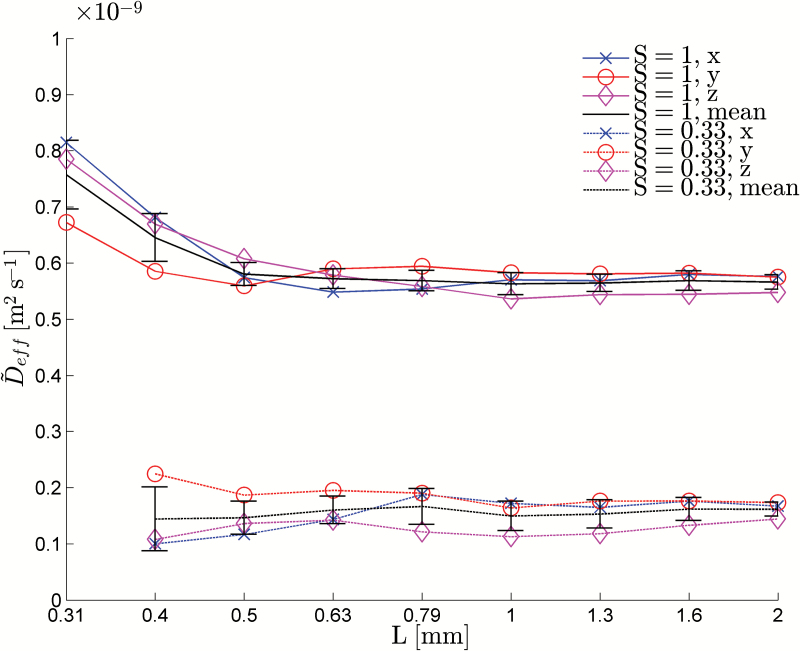
Effective diffusion coefficient as a function of *L*, the side length of the cube. The results were plotted for the saturated case (*S*=1), corresponding to the pore space being completely full of water, and partially saturated (*S*=0.33), corresponding to one-third of the pore space being occupied by water. As *L* increased, the saturated and unsaturated effective diffusion properties were seen to converge to a steady value corresponding to the bulk diffusion coefficient. (This figure is available in colour at *JXB* online.)

Before we considered the nutrient uptake properties of the plant root system, we first found the homogenized properties of the bulk soil. From the CT image (Fig. 1), we selected a cube of soil of side length *L*_*max*_=2mm. From this, we subsampled a series of geometries of size $L=2^{-n/3}L_{max}$ for *n*=0, 1,…8, i.e. we repeatedly halved the volume of bulk soil considered, and solved Eqns A31–A34 and A58 (Supplementary data A) to obtain ${\tilde{D}}_{eff}$. The key difficulty that arises in solving these equations is that they require the geometry to be periodic, i.e. composed of regularly repeating units. In reality, this is not the case. Hence, we have to impose periodicity. Following the method used by Tracy *et al.* (2015), we imposed periodicity by reflecting the geometry about the three coordinate axes. We note that this reflection is achieved mathematically through analysis of the symmetries of the problem rather than by physically copying the meshes. This is discussed in more detail in the Supplementary data and resulted in solving equations A35–A39. Computational resources ranged from 46 Gb across two nodes for 3min for the saturated case with *n*=8 to 900 Gb across 16 nodes for 45min for the more complex partially saturated case with *n*=0. As we increased the size of *L*, we found that the value of ${\tilde{D}}_{eff}$ converged to a fixed value once a sufficiently large subsample volume was included. Figure 4 shows these values for saturated and partially saturated soil. We see that for small *L* the effective diffusion coefficient is a function of *L*. However, as *L* increased, the value was seen to level off and become effectively independent of our choice of *L*. We interpreted this to mean that ${\tilde{D}}_{eff}$ had converged and would not change if *L* was increased further. As a measure of convergence, we assumed that ${\tilde{D}}_{eff}$ had converged if doubling the volume of soil considered, corresponding to increasing *n* by 1, caused a change in ${\tilde{D}}_{eff}$ that was smaller than 5% of its final value. The effective diffusion constant returned a converged value for n≤5, corresponding to *L*=0.63mm (Fig. 4). However, due to the symmetry reduction used in deriving Eqns A35–A39 (Supplementary data A), this side length must be doubled to obtain the true representative volume. Hence, the actual size of the representative volume was *L*=1.26mm. At this value, the computational resources used were 75 Gb across two nodes for 45min in the saturated case and 150 Gb across four nodes for 7min in the partially saturated case. The larger resource requirements were necessary for the partially saturated case due to the increased resolution needed to capture thin water films. We note that, although a representative side length of *L*=1.26mm may seem small, the soils used here were sieved to between two relatively close tolerances 1.00 and 1.68mm. Hence, these soils will be very homogeneous and we would expect the final soil packing to be close to an ideal sphere packing. The value of ${\tilde{D}}_{eff}$ was seen to converge to ${\tilde{D}}_{eff}\approx 0.56\times 10^{-9}{\text{m}}^{2}{\text{s}}^{-1}$ for the saturated soil and ${\tilde{D}}_{eff}\approx 0.15\times 10^{-9}{\text{m}}^{2}{\text{s}}^{-1}$ for the partially saturated soil. No data was plotted for the partially saturated case with *n*=8 because for samples this small, the air films and soil provided a complete barrier to diffusion and no effective diffusion coefficient could be calculated.

### Nutrient uptake properties

Using the values obtained for ${\tilde{D}}_{eff}$ at full and partial saturation, we next considered the uptake of nutrient by the plant root system shown in Fig. 4. We were interested in finding the size of the volume of soil about the root that we needed to consider geometry explicitly in order to accurately represent the uptake of phosphate by the root and root hairs. To determine the size of this volume, we considered a segment of angle $\tilde{\theta }$, height $\tilde{h}$, and radius ${\tilde{r}}_{b}$ centred on the root. The maximum radius considered was ${\tilde{r}}_{max}\;=1.76\;\text{mm}$, ${\tilde{\theta }}_{max}=\pi ,$ and ${\tilde{h}}_{max}=1.8\text{ mm.}$ The height, radius, and angle of the segment considered were increased from 20% of the maximum domain size in steps of 20% until the maximum was reached corresponding to a total of 125 simulations for each of the saturated and partially saturated cases. Computational requirements ranged from 15 Gb across two nodes for 15min in the simplest case to 300 Gb across 12 nodes for 16h in the most complex case.

**Fig. 5.**
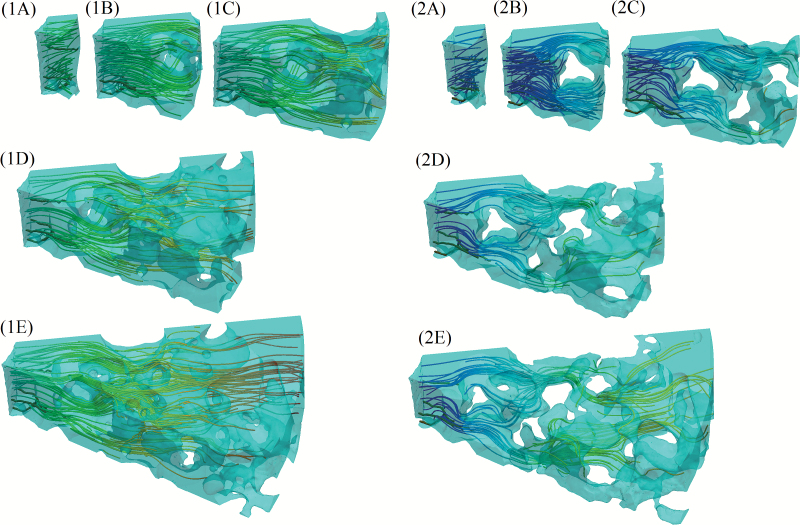
Typical plots obtained from simulation for the saturated case (1A–E). and the partially saturated case (2A–E). The five images in each case correspond to $\theta =0.2\;\theta_{max}=\pi /5,$ and $h=0.2h_{max}=0.36\;mm$ for (A) $r=0.2r_{max}=0.352\;mm\text{,}$ (B) $r=0.4r_{max}=0.704\;mm,$ (C) $r=0.6r_{max}=1.056\;mm\text{,}$ (D) $r=0.8r_{max}=1.408\;mm\text{,}$ and (E) $r=1.0r_{max}=1.76\;mm\text{.}$ The streamlines show the effective diffusion paths, with red corresponding to high nutrient concentration and blue corresponding to lower nutrient concentration. (This figure is available in colour at *JXB* online.)

**Fig. 6.**
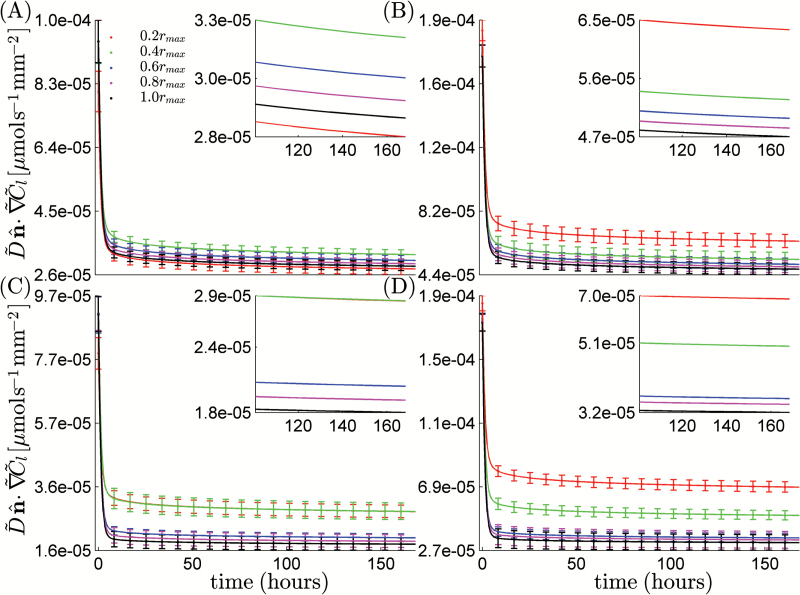
Log-linear plot of flux into the root hairs in the saturated case (A), the roots in the saturated case (B), the root hairs in the unsaturated case (C), and the roots in the unsaturated case (D). The lines show the average flux over a period of 1 week for the five different radii considered up to a maximum of *r*_*max*_=1.76mm. As *r* is increased, the uptake profile was seen to converge. The figure insets show a zoomed-in section of the same curve with error bars removed for clarity. (This figure is available in colour at *JXB* online.)

**Fig. 7.**
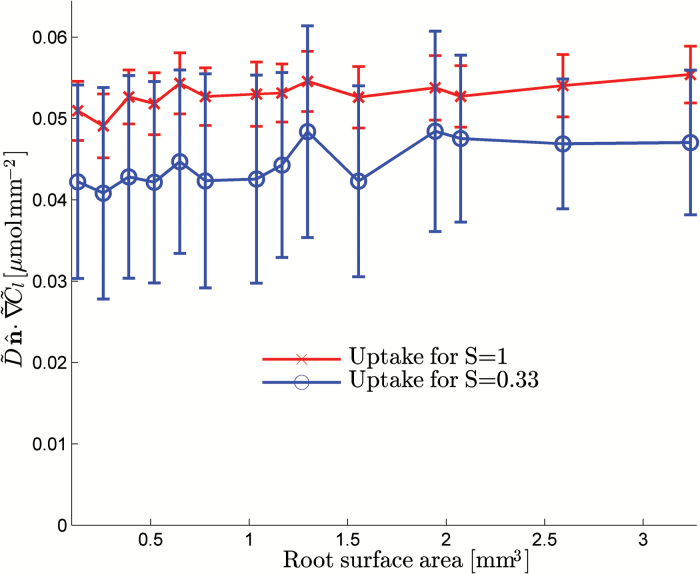
Total uptake into the root and root-hair system averaged over all time points. The plot shows the average uptake over the different radii considered with standard deviation error bars as a function of the root surface area. (This figure is available in colour at *JXB* online.)

Typical plots for the nutrient movement in the saturated and partially saturated cases are shown in Fig. 5 for a variety of ${\tilde{r}}_{b}$ values with the smallest $\tilde{\theta }$ and $\tilde{h}$ values used. Initially, we expected that there would be two convergence criteria. First, a sufficiently large root surface area would need to be considered in order for the number of root hairs involved in nutrient uptake to be representative, i.e. the density of root hairs in the subvolume is equal to the density of root measured density of root hairs. Secondly, the radius of the region considered must be sufficiently large that all the rhizosphere soil is captured. However, while there was some variance in the data with root surface area (see error bars in Fig. 6, and Fig. 7), it seemed that the main convergence criteria was that a sufficiently large value of ${\tilde{r}}_{b}$, i.e. the location of the outer rhizosphere–bulk soil boundary, was used. While this conclusion is somewhat surprising, it seems that only a relatively small value for $\tilde{\theta }$ and $\tilde{h}$ are required to capture enough of the root-hair structure that it is representative. On inspection, we saw that the ratio between the root-hair area and root area quickly settled to ≈0.5±0.05 for root-hair surface areas greater than 0.2mm^2^. Hence, for root surfaces areas above this value, the effective density of root-hair surface area did not change and the uptake properties were not expected to change significantly.

To check the convergence of the simulated phosphorous dynamics, we compared the total flux at the root and root-hair surfaces as a function of time for the linear uptake conditions. These data are presented in Fig. 6 for both saturated and partially saturated conditions for the root uptake and Fig. 7 for the root-hair uptake. The simulated nutrient uptake was seen to settle to a rate that was independent of ${\tilde{r}}_{b}$ for sufficiently large ${\tilde{r}}_{b}$. We assumed that the total uptake had converged once increasing ${\tilde{r}}_{b}$ produced a change in uptake of <5% of the final value. The radius at which this was seen to occur was ${\tilde{r}}_{b}=0.6\;{\tilde{r}}_{max}=1.1\text{ mm}$ for the saturated case and ${\tilde{r}}_{b}=0.8{\tilde{r}}_{max}=1.4\text{ mm}$ for the partially saturated case. In the saturated case at ${\tilde{r}}_{b}=0.2\;{\tilde{r}}_{max}$, the cumulative uptake of the root and root-hair surfaces was 58.8×10^–3^ μmol mm^–2^, and this converged to 50.0×10^–3^±10^–3^ μmol mm^–2^ for ${\tilde{r}}_{b}\geq 0.6{\;\tilde{r}}_{\text{max}}.$ In the partially saturated case, the cumulative uptake at ${\tilde{r}}_{b}=0.2\;{\tilde{r}}_{max}$ was 62.4×10^–3^ μmol mm^–2^. This converged to 31×10^–3^±10^–3^ μmol mm^–2^ for ${\tilde{r}}_{b}\geq 0.8{\;\tilde{r}}_{\text{max}}.$It is interesting at this point to compare our model with the zero flux approximation used by Keyes *et al.* (2013). In this case, the total uptake can easily be calculated as the total amount of phosphate available in the geometry at time $\tilde{t}=0$. For the largest saturated domain we have considered, this would be a total uptake of $5.54\times 10^{-4}\;\mu \text{mol }{\text{mm}}^{-2},$ which is significantly less than the uptake measured here of 50.0×10^–3^ μmol mm^–2^. We noted that the uptake by the root hairs converged in a diminishing oscillatory manner, i.e. the uptake first increased with ${\tilde{r}}_{b}$ before decaying to a steady state. This is because at the smallest value of ${\tilde{r}}_{b}$ the geometry does not contain the entire length of the root hairs. Hence, the root-hair uptake was noticeably lower than the cases of ${\tilde{r}}_{b}\geq 0.4{\;\tilde{r}}_{\text{max}}$. Once the full hair length was taken into account, the convergence was smooth and behaved the same way as the convergence of the root uptake.

As with the effective diffusion constant, we found that we did not need to consider a large amount of soil in order to obtain converged properties. Hence, we could observe that the radius required to capture the behaviour of the rhizosphere was dependent on the saturation of the fluid. In the fully saturated case, the radius simply needed to be large enough to be slightly greater than 100% of the root-hair length, i.e. 1.1mm. However, in the partially saturated case, a much larger radius (roughly double the root-hair length) was required to capture the corresponding effect on the nutrient motion. We observed that using this model we could parameterize mathematically simpler models such as the one developed by Roose *et al.* (2001). By using the effective diffusion constant and additional uptake provided by the root hairs to parameterize the model in Roose *et al.* (2001), we obtained a cumulative uptake of 50.0×10^–3^ μmol mm^–2^ using ${\tilde{D}}_{eff}=0.56\;\tilde{D}$ and ${\tilde{\lambda }}_{eff}=1.3\tilde{\lambda }$ for the uptake parameter in the saturated case. In the partially saturated case, we obtained a cumulative uptake of 31.2×10^–3^ μmol mm^–2^ using ${\tilde{D}}_{eff}=0.15\;\tilde{D}$ and ${\tilde{\lambda }}_{eff}=0.75\tilde{\lambda }$. In both cases, the model developed by Roose *et al.* (2001) agreed well with our predictions assuming the effective uptake parameter was a function of saturation. In the fully saturated case, the root hairs offered an increase in uptake of ~30%. In the partially saturated case, the effective uptake was decreased. This was in part due to the decrease in root and root-hair surface area that is in contact with water: 80% compared with the fully saturated case. Hence, while the root hairs considered in this geometry did increase root uptake when comparing image-based simulations, they did not significantly alter the uptake parameters in the conventional models.

### Root-hair growth

**Fig. 8.**
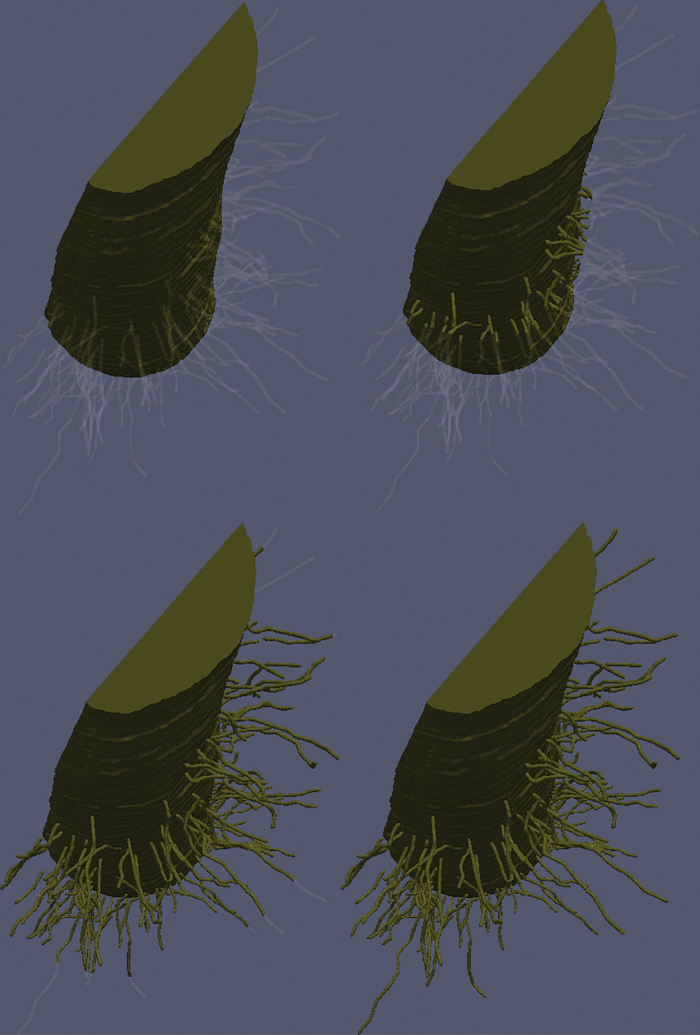
Root growth scenario for four different times. Top left shows the initial condition (0h), top right shows the hair growth after 16h, bottom left shows hair growth after 32h, and bottom right shows hair growth after 48h. (This figure is available in colour at *JXB* online.)

**Fig. 9.**
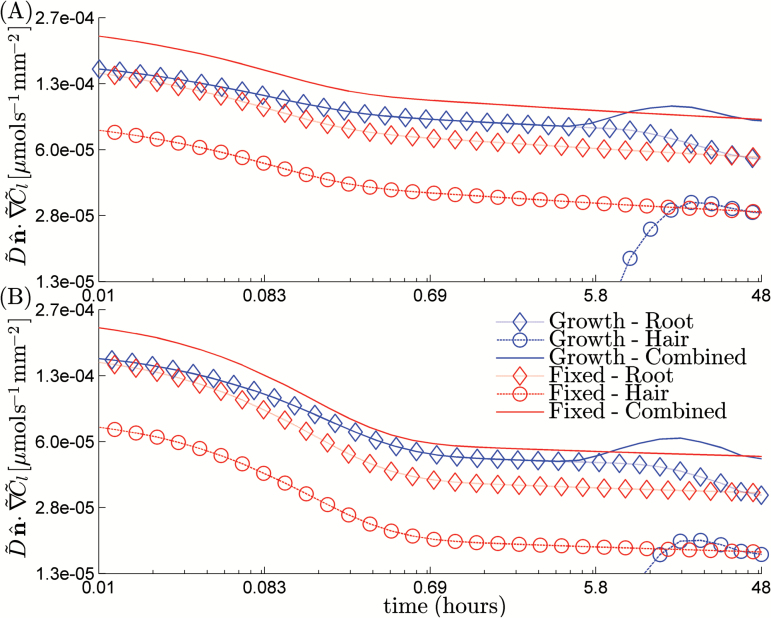
Flux at the root and hair surfaces for the root growth scenario in comparison with the fixed root-hair scenario for the saturated and partially saturated geometries (log scale). The combined uptake of the root and hair is also plotted. (This figure is available in colour at *JXB* online.)

We next considered the root-hair growth scenario. We neglect the geometrical growth of the root hairs and consider a growing region on which uptake occurs. We used a representative set of values for $\tilde{\theta }$, $\tilde{h}$, and ${\tilde{r}}_{b}$ for which the simulation had converged. Specifically, we took $\;\tilde{\theta }=0.6\;{\tilde{\theta }}_{max}$, $\tilde{h}=0.6{\tilde{h}}_{max},$ and ${\tilde{r}}_{b}=0.6{\tilde{r}}_{max}$ for the fully saturated case and $\tilde{\theta }=0.6\;{\tilde{\theta }}_{max}$, $\tilde{h}=0.6{\tilde{h}}_{max}$, and ${\tilde{r}}_{b}=0.8{\tilde{r}}_{max}$ for the partially saturated case. The root-hair growth is shown schematically in Fig. 8 for a range of different times. The hair-growth parameter ${\tilde{g}}_{r}=5\times 10^{-9}\text{m }{\text{s}}^{-1}$ was chosen to give the root hairs an effective growth period of 2 d, and the empirical parameter $\tilde{\alpha }=0.2\times 10^{6}{\text{m}}^{-1}$ was chosen such that ${\tilde{\alpha }}^{-1}$, i.e. the distance between the active and non-active sections of the root hair, was of length 5 μm. Alongside the uptake results for the saturated and partially saturated cases, we plotted the uptake results for the standard fixed root hairs case (Fig. 9). Interestingly, other than the magnitude of the flux, there is little qualitative difference between the saturated and partially saturated cases.

The main difference between the root-hair growth and the static root-hair scenario was observed towards the start of the growth period, between 0 and 48h. In the growth scenario, as expected, the uptake was dominated by the root for short times. However, as time progressed, the root hairs grew and provided the dominant contribution to nutrient uptake. Once the nutrients in the region immediately adjacent to the root had been taken up, the uptake in the growing root hair quickly settled to match the uptake rate for the fixed root hair case. After 48h when the root hairs were fully developed, the fixed and growing root-hair scenarios had identical uptake power. Hence, after 48h, the difference in cumulative uptake between the two scenarios was <1%. This suggested that the fixed root hair provides a good approximation for nutrient uptake, and that more detailed modelling of root-hair growth may not be necessary for timescales longer than 48h and shorter than the root-hair lifetime.

## Conclusions

In this study, we extended the image-based modelling of Keyes *et al.* (2013) to consider a large/infinite volume of soil around a single hairy root. The bulk soil properties were captured from an X-ray CT image-based geometry using the method of homogenization to transform the imaged geometry into a homogeneous medium with an effective diffusion constant based on the geometrical impedance offered by the soil. The bulk soil region was then patched onto the rhizosphere using a boundary condition that related the concentration at the surface of the rhizosphere to the flux into the rhizosphere. The advantage of image-based modelling of this type is that it can be used to answer specific questions on the movement of nutrients based on the soil geometry and root-hair morphology. This information can then be used to parameterize simplified models and gain an understanding of rhizosphere processes.

The method was tested for two different soil water saturation values by considering bulk and rhizosphere soil samples of different sizes, which were increased until the effective transport and uptake properties of the two regions were seen to converge. We found that the key criterion for convergence of nutrient uptake simulations was that a sufficiently large radius of soil about the root was considered. However, we emphasize that this is likely to be dependent on root-hair morphology, moisture content, and soil type. Hence, convergence checks should be carried out on a smaller scale for different geometries. It is interesting to note that the radius of the segment about the root needed for convergence to occur was dependent on the saturation considered. Specifically, we needed to consider a larger region of soil about the root for lower saturation values: 1.4mm of soil compared with the 1.1mm of soil for the saturated case. This observation was attributed to chemical effects present in the rhizosphere that cause variation in the fluid properties (Gregory, 2006). In our CT scans, these changes would be picked up as a geometrical variation either in the fluid location or the contact angle at the air–water interface. Hence, in the fully saturated case, we would not see these effects as the specific water location is neglected. By dividing the CT image into regions $\tilde{r}<0.2{\tilde{r}}_{max},$ $0.2{\tilde{r}}_{max}<\tilde{r}<0.4{\tilde{r}}_{max}$, $0.4{\tilde{r}}_{max}<\tilde{r}<0.6{\tilde{r}}_{max}$ $0.6{\tilde{r}}_{max}<\tilde{r}<0.8{\tilde{r}}_{max},$ and $0.8{\tilde{r}}_{max}<\tilde{r}<{\tilde{r}}_{max}$ and calculating the saturation in each case, we saw a noticeable decrease in saturation for $0.4{\tilde{r}}_{max}<\tilde{r}<0.6{\tilde{r}}_{max}$ (S≈0.5 compared with S>0.7 for all other regions). Therefore, in order to capture the geometric impedance created by this region, we require ${\tilde{r}}_{b}>0.6{\tilde{r}}_{max}$.

In agreement with the study by Keyes *et al.* (2013), we observed that in both cases the root hairs contributed less than the root surfaces to the nutrient uptake. However, this difference was small and, in terms of order of magnitude, both the hairs and the roots contributed equally to the nutrient uptake. A major advantage to this type of modelling is that these simulations can be used to parameterize existing models that may be computationally less challenging, for example that described by Roose *et al.* (2001). In this case, we see that the root hairs contribute approximately an extra 30% to the root uptake, an important consideration, which will increase the predictive ability of the model developed by Roose *et al.* (2001). Again, it may be that in poorly saturated soils, i.e. those with a moisture content much lower than 33%, there will be less water in contact with the root, and the root hairs, which can penetrate into the smaller pores within the soil, may provide a more significant contribution. Further investigation across a range of saturation values based on either imaging of samples at different water content or on application of a spatially explicit moisture content model such as the one developed in Daly and Roose (2015) would be needed to verify this.

Using the minimum values of $\tilde{h}$, *${\tilde{r}}_{b}$*, and $\tilde{\theta }$ obtained for the two cases, we considered the effect of pseudo root-hair growth on nutrient uptake, neglecting the geometrical and mechanical effects of root-hair growth. As the non-active root hairs are still geometrically represented in the domain, they may act to weakly impede the diffusion from the soil into the root itself. The root-hair growth modelling showed that, although there were differences in the ratio of root and hair uptake rates towards the start of the growth period, i.e. 2 d, on the long timescale, i.e. times greater than 2 d, the rate of uptake was unaffected by the growing root hairs (Fig. 9). This suggests that more detailed modelling of the root-hair growth might not yield different results, and the fixed root-hair approximation may provide a detailed enough picture for further investigation into the effects of root hairs.

The method developed here provides a powerful framework to study properties of the rhizosphere. We tested the model for a specific geometry obtained from X-ray CT. However, we emphasize that, whilst the reuslts obtained are suggestive of certain trends, they will be dependent on the precise soil type, water content and root-hair morphology studied. Hence, studying different root-hair distributions and soil structures will allow a greater understanding of how root hairs uptake nutrients to be developed. Further development of this method to couple a spatially explicit water model to the nutrient uptake will allow a similar understanding to be developed for water distribution and uptake.

## Supplementary data

Supplementary data are available at *JXB* online.

**Fig. S1** Schematic of the assay used for imaging.

**Data A.** Diffusion in bulk soil.

**Data B.** Resupply boundary condition.

Supplementary Data
