# Supplementary material for: Image-based modelling of nutrient movement in and around the rhizosphere
Source: J Exp Bot. 2016 Jan 5;67(4):1059–70. doi: 10.1093/jxb/erv544 (PMC4753851; doi:10.1093/jxb/erv544)
Supplement: Supplementary Data [file supp_67_4_1059__index.html]

Image-based modelling of nutrient movement in and around the rhizosphere — Image-based modelling of nutrient movement in and around the rhizosphere — Supplementary Data 

# Image-based modelling of nutrient movement in and around the rhizosphere

## Supplementary Data

Data files

- supplementary\_figure\_S1.pdf - Supplementary Data
