# Supplementary material for: Image-based modelling of nutrient movement in and around the rhizosphere
Source: J Exp Bot. 2016 Jan 5;67(4):1059–70. doi: 10.1093/jxb/erv544 (PMC4753851; doi:10.1093/jxb/erv544)
Supplement: Supplementary Data [file supp_erv544_supplementary_figure_S1.pdf]

Image based modeling of nutrient movement in and around the rhizosphere

Keith Daly, Samuel David Keyes, Shakil Masum, and Tiina Roose

Supplemental File

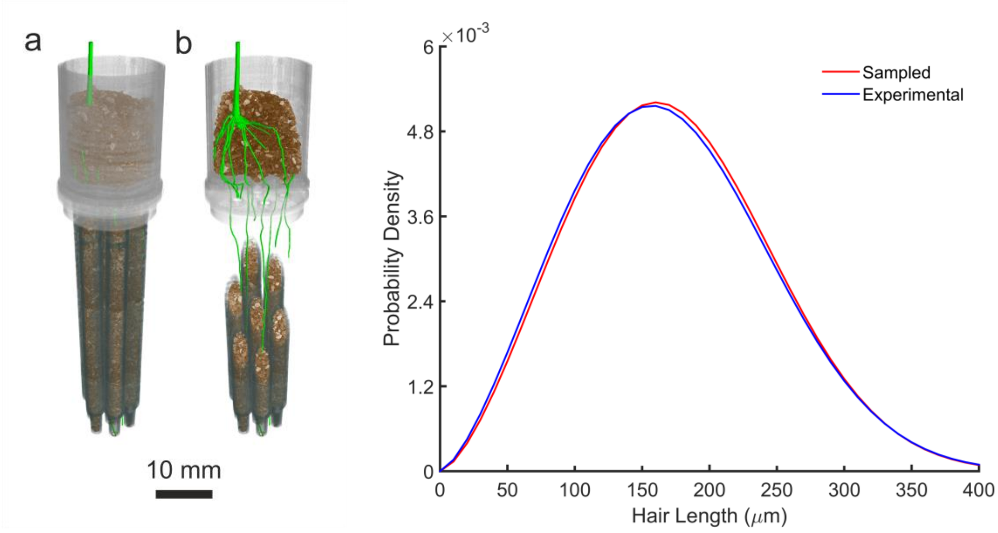

Supplementary Figure 1: Schematic of the assay used for imaging. a) The seed cup guides roots into an array of 7 soil-filled syringe barrels. B) In cutaway view, it is seen that individual barrels contain roots. The seed cup is removed, excising the roots, and barrels are then individually imaged using SRXCT.
